# Garnet-type Mn_3_Cr_2_(GeO_4_)_3_

**DOI:** 10.1107/S1600536812016832

**Published:** 2012-04-21

**Authors:** Christian Lipp, Sabine Strobel, Falk Lissner, Rainer Niewa

**Affiliations:** a Institut für Anorganische Chemie, Universität Stuttgart, Pfaffenwaldring 55, 70569 Stuttgart, Germany

## Abstract

Single crystals of garnet-type trimanganese(II) dichrom­i­um(III) tris­[orthogermanate(IV)], Mn^II^_3_Cr^III^_2_(GeO_4_)_3_, were obtained by utilizing a chemical transport reaction. Corres­ponding to the mineral garnet with the general formula *A* ^II^_3_ *B* ^III^_2_(SiO_4_)_3_, each of the four elements occupies only one crystallographically distinct position. Mn^2+^ occupies the respective *A* position (Wyckoff site 24*c*, site symmetry 2.22), being surrounded by eight O atoms that form a distorted cube [*d*(Mn—O) = 2.291 (2) and 2.422 (2) Å, 4× each], while Cr^3+^ on the *B* position (Wyckoff site 16*a*, site symmetry .-3.) is situated in a slightly distorted octa­hedron of six O^2−^ anions [*d*(Cr—O) = 1.972 (2) Å, 6×]. In addition, the O atoms on general site 96*h* form isolated [GeO_4_]^4−^ tetra­hedra with Ge^4+^ on site 24*d* [site symmetry -4..; *d*(Ge—O) = 1.744 (2) Å, 4×].

## Related literature

For general background to garnets, see: Geller (1967 ▶); Geusic *et al.* (1964 ▶); Menzer (1925 ▶, 1926 ▶, 1928 ▶); Nishikawa (1917 ▶); Novak & Gibbs (1971 ▶). For synthetic details, see: Binnewies *et al.* (2011 ▶); Pajączkowska & Majcher (1985 ▶, 1986 ▶); Pajączk­owska *et al.* (1986 ▶). For isotypic structures, see: Andrut & Wildner (2002 ▶); Belov *et al.* (1972 ▶); Fursenko (1981 ▶); Geller *et al.* (1960 ▶); Golosovskii *et al.* (1976 ▶); Lind & Geller (1969 ▶); Prandl (1973 ▶); Tauber *et al.* (1958*a* ▶,*b* ▶, 1961 ▶); Wildner & Andrut (2001 ▶).

## Experimental

### 

#### Crystal data

Mn_3_Cr_2_(GeO_4_)_3_
*M* *_r_* = 678.59
Cubic, 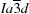

*a* = 12.0001 (3) Å
*V* = 1728.04 (7) Å^3^
*Z* = 8
Mo *K*α radiation
μ = 17.01 mm^−1^
*T* = 293 K
0.18 × 0.15 × 0.09 mm

#### Data collection

Nonius KappaCCD diffractometer
Absorption correction: numerical (*HABITUS*; Herrendorf & Bärnighausen, 1997 ▶) *T*_min_ = 0.063, *T*_max_ = 0.201
7635 measured reflections
277 independent reflections
265 reflections with *I* > 2σ(*I*)
*R*_int_ = 0.084

#### Refinement

*R*[*F* ^2^ > 2σ(*F* ^2^)] = 0.025
*wR*(*F* ^2^) = 0.068
*S* = 1.26
277 reflections
18 parameters
Δρ_max_ = 0.63 e Å^−3^
Δρ_min_ = −0.72 e Å^−3^

### 

Data collection: *COLLECT* (Nonius, 1998 ▶); cell refinement: *SCALEPACK* (Otwinowski & Minor, 1997 ▶); data reduction: *DENZO* (Otwinowski & Minor, 1997 ▶) and *SCALEPACK*; program(s) used to solve structure: *SHELXS97* (Sheldrick, 2008 ▶); program(s) used to refine structure: *SHELXL97* (Sheldrick, 2008 ▶); molecular graphics: *DIAMOND* (Brandenburg & Putz, 2012 ▶); software used to prepare material for publication: *publCIF* (Westrip, 2010 ▶).

## Supplementary Material

Crystal structure: contains datablock(s) i, global. DOI: 10.1107/S1600536812016832/wm2621sup1.cif

Structure factors: contains datablock(s) I. DOI: 10.1107/S1600536812016832/wm2621Isup2.hkl

Additional supplementary materials:  crystallographic information; 3D view; checkCIF report

## Figures and Tables

**Fig. 1.**
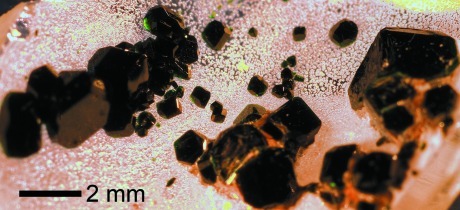
Dark green crystals of garnet-type MnII3CrIII2(GeO4)3 in the shape of rhombic dodecahedra, often with truncated edges. The photograph shows the crystals as they were grown after twelve days.

**Fig. 2.**
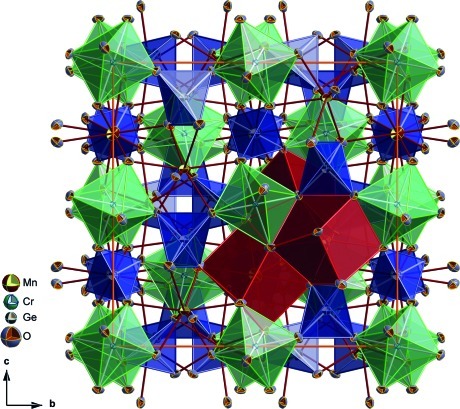
Overview of the structure of MnII3CrIII2(GeO4)3. For a better understanding only a group of three edge-connected distorted cubes [MnO8], representing the polyhedra about Mn2+, are drawn besides [CrO6] octahedra and [GeO4]4– tetrahedra (displacement ellipsoids are drawn at the 95% probability level).

**Fig. 3.**
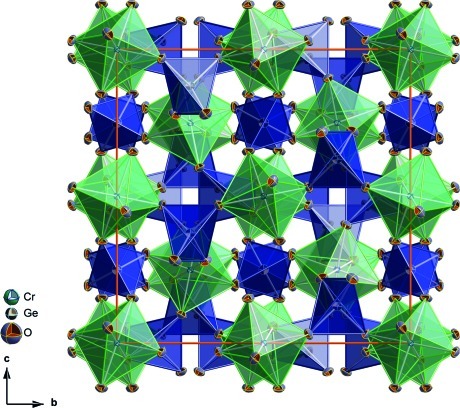
The network of vertex sharing [CrO6] octahedra and [GeO4]4– tetrahedra in the neso germanate Mn3Cr2(GeO4)3 (displacement ellipsoids are drawn at the 95% probability level).

## supplementary crystallographic information

### Comment

Garnet can be found among the very early attempts to solve crystal structures with the help of X-ray diffraction (Nishikawa, 1917), soon being followed by the understanding of the regular structure (Menzer, 1925, 1926) and the variability of garnet in a systematic way (Menzer, 1928; Geller, 1967; Novak & Gibbs, 1971). The variability of the garnet-type structure led for example to the well known Nd:YAG lasers (Y_3_Al_5_O_12_:Nd^3+^; Geusic *et al.*, 1964). A much more apparent variation is of course the replacement of Si by Ge (Tauber *et al.*, 1961; Geller, 1967), resulting in germanate garnets that are accessible *via* chemical transport reactions. The latter are an elegant way to grow crystals (Binnewies *et al.*, 2011) when other methods deliver only fine powders or require very high temperatures if the growth from melts is necessary.

The formation of garnet-type Mn_3_Cr_2_(GeO_4_)_3_ by chemical transport reactions was already successfully demonstrated for transporting agents like Cl_2_, CCl_4_, SCl_4_ and TeCl_4_ (Pajączkowska & Majcher, 1985, Pajączkowska *et al.* 1986; Mn_3_Fe_2_(GeO_4_)_3_: Pajączkowska & Majcher, 1986). While only 0.5 mm edge length as a maximum crystal size was stated when TeCl_4_ was used as a transporting agent, we achieved sizes of up to 4 mm within twelve days with the same agent. Quite interestingly Pajączkowska & Majcher (1985) determined only the unit-cell dimensions by using powder X-ray diffraction (*a* = 12.030 (1) Å), but not the structural details. The same germanate Mn_3_Cr_2_(GeO_4_)_3_ was already presented before, but each time again only by mentioning the unit-cell dimensions, being obtained by powder diffraction (Tauber *et al.*, 1958*a* (*a* = 12.027 Å); Tauber *et al.*, 1958*b* (*a* = 12.027 Å); Geller *et al.*, 1960 (*a* = 12.027 (3) Å); Belov *et al.*, 1972 (*a* = 12.028 (4) Å)). In addition some experiments on the magnetic properties have been conducted (Tauber *et al.*, 1958*b*; Belov *et al.*, 1972; Golosovskii *et al.* 1976), but again without the determination of structural details of Mn_3_Cr_2_(GeO_4_)_3_. Golosovskii *et al.* (1976) conducted neutron diffraction experiments and discussed the magnetic structure. They achieved results similar to the ones obtained for the spessartite-type germanate garnet Mn_3_Al_2_(GeO_4_)_3_ by Prandl (1973), thus finding confirmation for Mn^2+^ on site 24*i* where it is eightfold coordinated by oxygen.

The obtained dark green garnet-type Mn_3_Cr_2_(GeO_4_)_3_ (see Fig. 1) crystallizes isotypically with regular garnets. While Mn^2+^, Cr^3+^ and Ge^4+^ are located on the special sites 24*c*, 16*a* and 24*d*, oxygen is found on general site 96*h*. Manganese(II) is eightfold coordinated by oxygen in a distorted cube (Fig. 2) with Mn–O distances of 2.291 (2) and 2.422 (2) Å (4× each). These values are in good agreement with those reported for Mn_3_Fe_2_(GeO_4_)_3_ (2.303 (7) and 2.421 (7) Å; Lind & Geller, 1969). For additional comparison the analogous garnet Mn_3_Cr_2_(SiO_4_)_3_ would be of interest, but once more only the unit-cell parameters, determined by powder diffraction, are mentioned (*a* = 11.766 (2) Å; synthesis at 80 kbar/1273 K; Fursenko, 1981). The green color of Mn_3_Cr_2_(GeO_4_)_3_ may be taken as an indicator for the +III oxidation state of chromium. Oxygen surrounds chromium(III) in a slightly distorted octahedron (Fig. 3) with *d*(Cr—O) = 1.972 (2) Å (6×), whereas 1.9942 (6) Å is reported for chromium on the same position in synthetic uvarovite (Ca_3_Cr_2_(SiO_4_)_3_; Andrut & Wildner, 2002; for natural uvarovite, see: Wildner & Andrut, 2001). Due to the slight distortion, the (O—Cr—O) angles within the [CrO_6_] octahedra deviate from 90°, *viz* 86.33 (8)° and 93.67 (8)°. Completing the description of the cation environments, Ge^4+^ is situated in slightly elongated tetrahedra of four O^2–^ anions where *d*(Ge—O) = 1.744 (2) Å, and the angles (O—Ge—O) are found to be 99.02 (14)° (2×) and 114.93 (7)° (4×). This is again similar to the values detected for Mn_3_Fe_2_(GeO_4_)_3_ (*d*(Ge—O) = 1.766 (7) Å, (O—Ge—O) = 98.8 (5)° (2×) and 115.1 (3)° (4×); Lind & Geller, 1969). Keeping in mind only a single site is occupied by oxygen, the structure of garnets might be described as two interpenetrating networks in Mn_3_Cr_2_(GeO_4_)_3_, the first made up of edge sharing [CrO_6_] octahedra and [GeO_4_]^4–^ tetrahedra (Fig. 2 & 3), while the second consists of groups of three edge sharing [MnO_8_] polyhedra (see Fig. 2), with each distorted [MnO_8_] cube being part of two of these units of three polyhedra. Therefore, each [MnO_8_] polyhedron shares four edges with four [MnO_8_] polyhedra, four edges with four [CrO_6_] octahedra, and two edges and four vertices with six [GeO_4_]^4–^ tetrahedra.

### Experimental

MnO, Cr_2_O_3_ and GeO_2_ (molar ratios of 3:1:3; MnO (Riedel-de Haën, *rein*): 212.8 mg, Cr_2_O_3_ (Riedel-de Haën, *rein*): 152.0 mg, GeO_2_ (ChemPur, 99.999%): 313.8 mg) and the transporting agent TeCl_4_ (Alfa-Aesar, 99.9%; 8.5 mg ml^-1^) were brought into a fused silica ampule (length: 120 mm, inner diameter: 16 mm) and sealed under vacuum. The following transport reaction was conducted within twelve days in a two-zone tube furnace with a temperature gradient of 70 K (*T*_2_ = 1293 K, *T*_1_ = 1223 K, with the transport direction *T*_2_→*T*_1_). Subsequent to the cooling to a temperature of 673 K within five hours, ambient temperature was reached by quenching in water. The obtained crystals had a size of up to 4 mm and shapes that are typically observed in garnets, *i.e.* derived from rhombic dodecahedra with often truncated edges. Larger crystals appear virtually black and opaque while smaller ones are transparent and dark green in color (see Fig. 1).

### Crystal data

**Table:**

Mn_3_Cr_2_(GeO_4_)_3_	*D*_x_ = 5.217 Mg m^−^^3^
*M**_r_* = 678.59	Mo *K*α radiation, λ = 0.71073 Å
Cubic, *I**a*3*d*	Cell parameters from 6052 reflections
Hall symbol: -I 4bd 2c 3	θ = 0.4–40.3°
*a* = 12.0001 (3) Å	µ = 17.01 mm^−^^1^
*V* = 1728.04 (7) Å^3^	*T* = 293 K
*Z* = 8	Truncated rhombic dodecahedron, dark green
*F*(000) = 2520	0.18 × 0.15 × 0.09 mm

### Data collection

**Table:**

Nonius KappaCCD diffractometer	277 independent reflections
Radiation source: fine-focus sealed tube	265 reflections with *I* > 2σ(*I*)
Graphite monochromator	*R*_int_ = 0.084
ω scans	θ_max_ = 33.1°, θ_min_ = 4.2°
Absorption correction: numerical (*HABITUS*; Herrendorf & Bärnighausen, 1997)	*h* = −18→16
*T*_min_ = 0.063, *T*_max_ = 0.201	*k* = −18→15
7635 measured reflections	*l* = −17→15

### Refinement

**Table:**

Refinement on *F*^2^	Primary atom site location: structure-invariant direct methods
Least-squares matrix: full	Secondary atom site location: difference Fourier map
*R*[*F*^2^ > 2σ(*F*^2^)] = 0.025	*w* = 1/[σ^2^(*F*_o_^2^) + (0.0115*P*)^2^ + 25.1758*P*] where *P* = (*F*_o_^2^ + 2*F*_c_^2^)/3
*wR*(*F*^2^) = 0.068	(Δ/σ)_max_ < 0.001
*S* = 1.26	Δρ_max_ = 0.63 e Å^−^^3^
277 reflections	Δρ_min_ = −0.72 e Å^−^^3^
18 parameters	Extinction correction: *SHELXL97* (Sheldrick, 2008), Fc^*^=kFc[1+0.001xFc^2^λ^3^/sin(2θ)]^-1/4^
0 restraints	Extinction coefficient: 0.0031 (2)

### Special details

**Table:**

Geometry. All e.s.d.'s (except the e.s.d. in the dihedral angle between two l.s. planes) are estimated using the full covariance matrix. The cell e.s.d.'s are taken into account individually in the estimation of e.s.d.'s in distances, angles and torsion angles; correlations between e.s.d.'s in cell parameters are only used when they are defined by crystal symmetry. An approximate (isotropic) treatment of cell e.s.d.'s is used for estimating e.s.d.'s involving l.s. planes.
Refinement. Refinement of *F*^2^ against ALL reflections. The weighted *R*-factor *wR* and goodness of fit *S* are based on *F*^2^, conventional *R*-factors *R* are based on *F*, with *F* set to zero for negative *F*^2^. The threshold expression of *F*^2^ > σ(*F*^2^) is used only for calculating *R*-factors(gt) *etc*. and is not relevant to the choice of reflections for refinement. *R*-factors based on *F*^2^ are statistically about twice as large as those based on *F*, and *R*- factors based on ALL data will be even larger.

### Fractional atomic coordinates and isotropic or equivalent isotropic displacement parameters (Å^2^)

**Table:**

	*x*	*y*	*z*	*U*_iso_*/*U*_eq_	
Mn	0.1250	0.0000	0.2500	0.0080 (3)	
Cr	0.0000	0.0000	0.0000	0.0023 (3)	
Ge	0.3750	0.0000	0.2500	0.0045 (2)	
O	0.03061 (17)	0.05245 (18)	0.65268 (17)	0.0065 (4)	

### Atomic displacement parameters (Å^2^)

**Table:**

	*U*^11^	*U*^22^	*U*^33^	*U*^12^	*U*^13^	*U*^23^
Mn	0.0039 (4)	0.0100 (3)	0.0100 (3)	0.000	0.000	0.0019 (3)
Cr	0.0023 (3)	0.0023 (3)	0.0023 (3)	0.00006 (17)	0.00006 (17)	0.00006 (17)
Ge	0.0036 (3)	0.0050 (3)	0.0050 (3)	0.000	0.000	0.000
O	0.0068 (9)	0.0082 (9)	0.0044 (8)	0.0016 (7)	−0.0018 (7)	−0.0006 (7)

### Geometric parameters (Å, º)

**Table:**

Mn—O^i^	2.291 (2)	Cr—O^xi^	1.972 (2)
Mn—O^ii^	2.291 (2)	Cr—O^xii^	1.972 (2)
Mn—O^iii^	2.291 (2)	Cr—O^ii^	1.972 (2)
Mn—O^iv^	2.291 (2)	Ge—O^xiii^	1.744 (2)
Mn—O^v^	2.422 (2)	Ge—O^iii^	1.744 (2)
Mn—O^vi^	2.422 (2)	Ge—O^i^	1.744 (2)
Mn—O^vii^	2.422 (2)	Ge—O^xiv^	1.744 (2)
Mn—O^viii^	2.422 (2)	O—Ge^xv^	1.744 (2)
Cr—O^viii^	1.972 (2)	O—Cr^x^	1.972 (2)
Cr—O^ix^	1.972 (2)	O—Mn^iv^	2.291 (2)
Cr—O^x^	1.972 (2)	O—Mn^xvi^	2.422 (2)
			
O^i^—Mn—O^ii^	112.61 (10)	O^v^—Mn—Ge^xvii^	97.88 (5)
O^i^—Mn—O^iii^	70.78 (10)	O^vi^—Mn—Ge^xvii^	82.12 (5)
O^ii^—Mn—O^iii^	160.86 (10)	O^vii^—Mn—Ge^xvii^	82.12 (5)
O^i^—Mn—O^iv^	160.86 (10)	O^viii^—Mn—Ge^xvii^	97.88 (5)
O^ii^—Mn—O^iv^	70.78 (10)	O^viii^—Cr—O^ix^	180.00 (17)
O^iii^—Mn—O^iv^	112.61 (10)	O^viii^—Cr—O^x^	93.67 (8)
O^i^—Mn—O^v^	94.58 (6)	O^ix^—Cr—O^x^	86.33 (8)
O^ii^—Mn—O^v^	124.69 (4)	O^viii^—Cr—O^xi^	93.67 (8)
O^iii^—Mn—O^v^	72.34 (8)	O^ix^—Cr—O^xi^	86.33 (8)
O^iv^—Mn—O^v^	69.78 (10)	O^x^—Cr—O^xi^	86.33 (8)
O^i^—Mn—O^vi^	124.69 (4)	O^viii^—Cr—O^xii^	86.33 (8)
O^ii^—Mn—O^vi^	94.58 (6)	O^ix^—Cr—O^xii^	93.67 (8)
O^iii^—Mn—O^vi^	69.78 (10)	O^x^—Cr—O^xii^	93.67 (8)
O^iv^—Mn—O^vi^	72.34 (8)	O^xi^—Cr—O^xii^	180.00 (17)
O^v^—Mn—O^vi^	108.41 (10)	O^viii^—Cr—O^ii^	86.33 (8)
O^i^—Mn—O^vii^	69.78 (10)	O^ix^—Cr—O^ii^	93.67 (8)
O^ii^—Mn—O^vii^	72.34 (8)	O^x^—Cr—O^ii^	180.00 (17)
O^iii^—Mn—O^vii^	124.69 (4)	O^xi^—Cr—O^ii^	93.67 (8)
O^iv^—Mn—O^vii^	94.58 (6)	O^xii^—Cr—O^ii^	86.33 (8)
O^v^—Mn—O^vii^	73.85 (10)	O^xiii^—Ge—O^iii^	114.93 (7)
O^vi^—Mn—O^vii^	164.23 (10)	O^xiii^—Ge—O^i^	114.93 (7)
O^i^—Mn—O^viii^	72.34 (8)	O^iii^—Ge—O^i^	99.02 (14)
O^ii^—Mn—O^viii^	69.78 (10)	O^xiii^—Ge—O^xiv^	99.02 (14)
O^iii^—Mn—O^viii^	94.58 (6)	O^iii^—Ge—O^xiv^	114.93 (7)
O^iv^—Mn—O^viii^	124.69 (4)	O^i^—Ge—O^xiv^	114.93 (7)
O^v^—Mn—O^viii^	164.23 (10)	O^xiii^—Ge—Mn	130.49 (7)
O^vi^—Mn—O^viii^	73.85 (10)	O^iii^—Ge—Mn	49.51 (7)
O^vii^—Mn—O^viii^	108.41 (10)	O^i^—Ge—Mn	49.51 (7)
O^i^—Mn—Ge	35.39 (5)	O^xiv^—Ge—Mn	130.49 (7)
O^ii^—Mn—Ge	144.61 (5)	O^xiii^—Ge—Mn^xviii^	49.51 (7)
O^iii^—Mn—Ge	35.39 (5)	O^iii^—Ge—Mn^xviii^	130.49 (7)
O^iv^—Mn—Ge	144.61 (5)	O^i^—Ge—Mn^xviii^	130.49 (7)
O^v^—Mn—Ge	82.12 (5)	O^xiv^—Ge—Mn^xviii^	49.51 (7)
O^vi^—Mn—Ge	97.88 (5)	Ge^xv^—O—Cr^x^	128.90 (11)
O^vii^—Mn—Ge	97.88 (5)	Ge^xv^—O—Mn^iv^	95.10 (9)
O^viii^—Mn—Ge	82.12 (5)	Cr^x^—O—Mn^iv^	103.54 (9)
O^i^—Mn—Ge^xvii^	144.61 (5)	Ge^xv^—O—Mn^xvi^	122.92 (10)
O^ii^—Mn—Ge^xvii^	35.39 (5)	Cr^x^—O—Mn^xvi^	99.02 (8)
O^iii^—Mn—Ge^xvii^	144.61 (5)	Mn^iv^—O—Mn^xvi^	102.43 (8)
O^iv^—Mn—Ge^xvii^	35.39 (5)		

Symmetry codes: (i) *x*+1/4, −*z*+3/4, −*y*+1/4; (ii) −*x*, *y*, *z*−1/2; (iii) *x*+1/4, *z*−3/4, *y*+1/4; (iv) −*x*, −*y*, −*z*+1; (v) *z*−1/2, *x*, −*y*+1/2; (vi) −*z*+3/4, *y*−1/4, −*x*+1/4; (vii) −*z*+3/4, −*y*+1/4, *x*+1/4; (viii) *z*−1/2, −*x*, *y*; (ix) −*z*+1/2, *x*, −*y*; (x) *x*, −*y*, −*z*+1/2; (xi) −*y*, −*z*+1/2, *x*; (xii) *y*, *z*−1/2, −*x*; (xiii) −*x*+1/2, *y*, −*z*+1; (xiv) −*x*+1/2, −*y*, *z*−1/2; (xv) −*x*+1/2, −*y*, *z*+1/2; (xvi) −*y*, *z*, *x*+1/2; (xvii) *x*−1/2, *y*, −*z*+1/2; (xviii) *x*+1/2, *y*, −*z*+1/2.
